# The impact of COVID-19 lockdown on acute trauma patients seen at the National Hospital Trauma Centre Abuja, Nigeria

**DOI:** 10.11604/pamj.2021.38.414.28431

**Published:** 2021-04-30

**Authors:** Onyedika Godfrey Okoye, Oluwole Olayemi Olaomi, Usman Adamu Gwaram, Kanati Dennis Apollo

**Affiliations:** 1 Trauma Centre, National Hospital Abuja, Abuja, Nigeria

**Keywords:** COVID-19, lockdown, acute, trauma

## Abstract

**Introduction:**

trauma is the leading cause of mortality in individuals less than 45 years. The principles of Advanced Trauma Life Support (ATLS) which is used around the world in resuscitation of trauma patients have been considered to be safe. However, the outbreak of corona virus disease 2019 (COVID-19) has affected the processes and characteristics of acute trauma patients seen around the world. This study is intended to determine the impact of COVID-19 lockdown on the acute trauma patients seen in a Nigerian trauma centre.

**Methods:**

this is a cross-sectional observational study of trauma patients seen in the resuscitation room of the National Hospital trauma centre in Abuja, Nigeria, from 24^th^ February,2020 to 3^rd^ May, 2020. The participants were consecutive acute trauma patients who were grouped into two: five weeks preceding total lockdown and five weeks of total lockdown. Statistical analysis was done using the statistical package for social sciences (SPSS) version 24.0 while results were presented in tables and a figure.

**Results:**

a total of 229 patients were recruited into the study with age range 1 to 62 years, mean age of 28 ± 13 and male to female ratio of 3.87. The patient volume reduced by 41.31% during the lockdown. Though motor vehicular crash (MVC) was the predominant mechanism of injury in both groups making up 37.65% and 23.88% respectively, penetrating assault was more during the lockdown period (17.91% versus 6.17%). The lockdown was further associated with more delayed presentation (52.24% versus 48.15%), more referrals (53.73% versus 32.72%), less severe injury score (29.6% versus 56.7%) and no death in the resuscitation room (0% versus 1.85%).

**Conclusion:**

despite the reduction in the volume of trauma presentations by 41.31%, patients got the required care with less mortality. Efforts should be directed at sustaining access to acute trauma care in all circumstances to reduce preventable trauma deaths.

## Introduction

Trauma remains the leading cause of death in individuals less than 45 years [1]. It is still a public health issue currently accounting for up to 18% of the global burden of diseases [2, 3]. Management of major trauma is protocol driven around the world. Most protocols are in line with the popular Advanced Trauma Life Support (ATLS) principles [4]. These principles have been adjudged to be safe and consistent even in the presence of infectious diseases while employing standard precautions. Recall that the novel corona virus disease (COVID-19) outbreak was first reported in December 2019 with the first patient hospitalized in the city of Wuhan, China [5]. It was further declared a public health emergency of international concern [6] on January 30^th^, 2020 and a pandemic [7] on March 11^th^, 2020 by the World Health Organization (WHO). Cases continue to be reported in varying proportions in different parts of the world with attendant mortalities.

COVID-19 is caused by the severe acute respiratory syndrome coronavirus 2 (SARS-CoV-2). The microbial pathogenesis results from host-microbial interaction with attendant host damage from microbial trait or the host immune response or both [8]. Clinical symptoms range from a mild upper respiratory tract-like illness to a life threatening acute respiratory acute respiratory syndrome [9], leading to compromised oxygenation by pulmonary inflammation. The virus damages the infected cells and triggers the production of proinflammatory cytokines which elicit inflammation that damages host cells and tissues locally and at a distance [10]. Neurological mechanisms and thromboembolism are also contributory factors [11, 12].

The COVID surge collaborative recommended thresholds for surgery should be heightened after analyzing an international multi centre cohort of patients who had any kind of surgery with a perioperative diagnosis of COVID-19 [13]. As cases of COVID-19 begin to surge at different trauma centres; changes in the delivery of direct patient care to injured patients require modification [14]. Many trauma centres have adopted more restrictive transfusion practices [15]. Some centres have limited the use of emergency department resuscitative thoracotomy, departing from standard indications [16]. Many hospitals are restricting early tracheostomy due to the perceived risk of incidental viral transmission [17]. Trauma experts believe that all efforts should be made to prevent trauma centres from becoming collateral damage in the fight against COVID-19 [18]. Introduction of lockdown measures in the United Kingdom led to a reported fall of 30 percent in overall emergency admissions [19]. A fall in trauma volume was also observed in Italy but with higher injury severity [20]. In general, the surge in acute ill patients from COVID-19 infections put a significant stress on the already overwhelmed health care system globally [21]. For purpose of emphasis, it is imperative that local acute trauma resuscitation protocols are modified to reflect the changes brought about by this pandemic [22]. This study is intended to compare the pattern of acute trauma patients seen in a dedicated Nigerian trauma centre before and during COVID-19 total lockdown in the country with a view of ascertaining the influence of the lockdown, if any on the patient characteristics.

## Methods

**Study design:** this is a cross-sectional observational study of patients seen in the resuscitation room of the trauma centre of National Hospital Abuja.

**Setting:** the study was done in national trauma centre, a level 1 dedicated trauma centre located in National Hospital Abuja, Nigeria. This centre is a referral centre for major trauma patients within and outside Abuja and serves a population of over three million people.

**Participants:** all acute trauma patients (presenting within the first 72 hours of their injury as per our existing trauma protocol) who were seen in the resuscitation room during the study period were recruited. The participants were divided into two groups: group A were patients seen in the five weeks period preceding the national total lockdown in Nigeria from 24^th^ February to 29^th^ March 2020 while group B were patients seen during the five weeks period of total lockdown from 30^th^ March to 3^rd^ May 2020.

**Variables:** information on their demographics including age and sex; mechanism of injury, mode of transportation, accompanying person, referral status, revised trauma score, injury severity score, diagnosis, disposition from the resuscitation room and final outcome were retrieved and subsequently analyzed.

**Data sources:** data comprising variables mentioned above was retrieved from the trauma registry kept by the trauma centre. Our trauma registry is 32 parameters excel spreadsheet with drop down options where all trauma patients´ information is recorded and updated every 24 hours. Some specific missing information in some cases was completed from the patient case note. Patients with incomplete data despite case notes information were excluded from the study.

**Bias:** potential sources of bias were addressed by ensuring equal duration of study for both groups of participants and involving an independent statistician for data analysis.

**Study size/sampling technique:** a total of 229 patients were recruited into the study using consecutive sampling method. These patients included 162 and 67 from group A and B periods respectively. The sample size was a reflection of all eligible patients (injuries 72 hours duration or less) who presented within the study period while the study period was determined by the period of government imposed total lock down in Nigeria.

**Statistical methods:** data recording, processing and analysis were done using statistical package for social sciences (SPSS) version 24.0. Univariate analysis of data on socio-demographic characteristics and functional outcomes was presented by use of measures of distribution, like frequency distribution tables, central tendency (mean, median and mode) and dispersions (range and standard deviation). As part of baseline analysis, the findings in group A and B patients were compared and subjected to statistical analysis including test of significance. Chi Square test (x^2^) and likelihood ratios (LR) were used to test the statistical association between/among the distribution on both sides of the study group. A value of P < 0.05 was considered significant. Frequency tables and charts were used where necessary to present the results.

**Ethical consideration:** ethical clearance was obtained from the institution review board of National Hospital Abuja, Nigeria.

## Results

A total of 229 patients were recruited into this study; 162 in the first five weeks preceding total lockdown and 67 in the second five weeks during the total lockdown, representing a 41.35% reduction in the patient volume during the lockdown period. Males were more affected than females in both periods with male to female ratio of 3.87. The mean age of the patients was 28 ± 13. The most frequent age groups were 21-30 years (38.27%), 31-40 years (22.84%) and 11-20 years (16.67%) before the lockdown; and 21-30 years, 31-40 years both accounting for 28.36% and less than 10 years (17.91%) during the lockdown. The least involved age group in both periods was more than 50 years group accounting for only 3.70% and 5.97% respectively (Table 1). While slightly more patients (51.85%) presented in less than two hours before the lockdown, slightly more patients (52.39%) presented after two hours during the lockdown.

**Table 1:** socio-demographic characteristics of the respondents presenting before and during the lockdown

Variables	Period of Presentation (%)		Statistics
	Before Lockdown	During Lockdown	
**Age groups (in years)**			
≤ 10	15 (9.26)	12 (17.91)	χ^2^ = 7.499
11 – 20	27 (16.67)	6 (8.96)	df = 5
21 – 30	62 (38.27)	19 (28.36)	p = 0.186
31 – 40	37 (22.84)	19 (28.36)	
41 – 50	15 (9.26)	7 (10.45)	
>50	6 (3.70)	4 (5.97)	
**Sex**			χ^2^ = 0.202
Male	130 (80.25)	52 (77.61)	df = 1
Female	32 (19.75)	15 (22.39)	p = 0.653

The most frequent mechanisms of injury were motor vehicular crash (MVC) (37.65%), motor bike crash (MBC) (19.75%) and domestic accident (8.64%) before the lockdown while MVC (23.88%), penetrating assault (17.91%) and MBC (13.43%) were more frequent during the lockdown. The least frequent mechanism of trauma was pedestrian vehicular crash (PVC) representing 5.56 and 4.48% respectively. The proportion of patients who were referred before the lockdown (32.64%) were nearly half of those who were not referred (67.28%); while in the lockdown period, slightly more patients (53.73%) were referred. Majority of injuries occurred in both the city and suburb at equal amount (43.83%) before the lockdown while most of them occurred in the suburb (52.24%) during the lockdown period (Table 2).

**Table 2:** conditions surrounding the injuries of respondents who presented before and during the lockdown

Variables	Period of Presentation (%)		Statistics
	Before Lockdown	During Lockdown	
**Duration**			χ^2^ = 0.317
≤ 2 hours	84 (51.85)	32 (47.71)	df = 1
>2 hours	78 (48.15)	35 (52.39)	p =0.573
**Mechanism of Injury**			
Blunt assault	13 (8.02)	8 (11.94)	χ^2^ = 12.941
Penetrating assault	10 (6.17)	12 (17.91)	df = 7
Burns	12 (7.41)	6 (8.96)	p = 0.074
Domestic	14 (8.64)	4 (5.97)	
Falls	11 (6.79)	8 (11.94)	
MBC	32 (19.75)	9 (13.43)	
MVC	61 (37.65)	16 (23.88)	
PVC	9 (5.56)	3 (4.48)	
**Referral status**			χ^2^ = 8.810
Referred	53 (32.64)	36 (53.73)	df = 1
Not referred	109 (67.28)	31 (46.27)	p = 0.003*
**Mode of transport**			
Ambulance	14 (8.64)	14 (20.90)	χ^2^ = 7.197
Commercial vehicle	14 (8.64)	7 (10.45)	df = 3
Private vehicle	83 (51.23)	29 (43.28)	p = 0.066
Security vehicle	51 (31.48)	17 (25.37)	
**Brought in by**			
Assailant	7 (4.32)	1 (1.49)	LR = 11.476
Colleague(s)	5 (3.09)	8 (11.94)	df = 6
Family	63 (38.89)	29 (43.28)	p = 0.075
Nurse/ doctor	12 (7.41)	6 (8.96)	
Passers by	13 (8.02)	2 (2.99)	
Security personnel	54 (33.33)	20 (29.85)	
Self	8 (4.94)	1 (1.49)	
**Location**			
City	71 (43.83)	28 (41.79)	χ^2^ = 2.608
Outside Abuja	20 (12.35)	4 (5.97)	df = 2
Suburb	71 (43.83)	35 (52.24)	p = 0.271
**Site**			
Home	28 (17.28)	17 (25.37)	LR = 4.553
Recreation centre	4 (2.47)	0 (0.00)	df = 3
Road	115 (70.99)	45 (67.16)	p = 0.208
Workplace	15 (9.26)	5 (7.46)	

* Statistically significant LR – Likelihood ratio used because more than 25% of the expected values had values less than 5

Soft tissue injuries (26.54%) followed by traumatic brain injury (25.92%) and fractures/dislocations (14.20%) constituted most of the diagnoses before the lockdown while traumatic brain injury (25.37%) followed by fractures/dislocations (16.42%) and polytrauma (16.42%) were the commonest diagnoses during the total lockdown. Majority of the injuries felt into the revised trauma score (RTS) of 12; both before (80.25%) and during (67.16%) the lockdown. Injury severity score (ISS) of 75 was recorded only in two patients before the lockdown and none of the patients during the lockdown era (Table 3).

**Table 3:** diagnosis and severity grading of respondents who presented before and during the lockdown

Variables	Period of presentation (%)		Statistics
	Before Lockdown	During Lockdown	
**Diagnosis**			
Soft tissue injury	43 (26.54)	10 (14.93)	LR = 14.182
Abdominal injury	8 (4.94)	2 (2.99)	df = 9
Chest injury	4 (2.47)	5 (7.46)	p = 0.116
TBI	42 (25.93)	17 (25.37)	
Burns	12 (7.41)	6 (8.96)	
Fractures and dislocations	23 (14.20)	11 (16.42)	
Maxillofacial injuries	8 (4.94)	1 (1.49)	
Neck injury	4 (2.47)	3 (4.48)	
Ocular injury	7 (4.32)	1 (1.49)	
Poly-trauma	11 (6.79)	11 (16.42)	
**RTS categories**			
3 - 10	11 (6.79)	13 (19.40)	χ^2^ = 8.264
11	21 (12.96)	9 (13.43)	df = 2
12	130 (80.25)	45 (67.16)	p = 0.0016*
**ISS categories**			
1 - 15	114 (70.37)	29 (43.28)	LR = 16.932
16 - 74	46 (28.40)	38 (56.72)	df = 2
75	2 (1.23)	0 (0.00)	p < 0.001*

* Statistically significant LR - Likelihood ratio used because more than 25% of the expected values had values less than 5 RTS - Revised Trauma Score, ISS - Injury Severity Score

**Figure 1:**
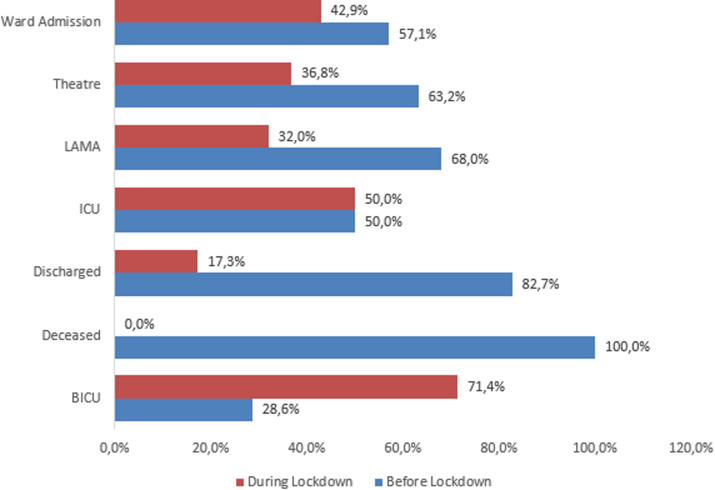
disposition from the resuscitation room among patients presenting before and during the lockdown

Figure 1 which compared the disposition of the patients from the resuscitation room revealed higher percentages for ward admissions, operating theatre cases, those who left against medical advice (LAMA) and those who died before the lockdown. However, there was equal intensive care unit (ICU) admission and more burns ICU admission during the lockdown. In all, the differences seen among the distribution of the variables before and during lockdown are statistically significant (P=0.001).

## Discussion

**Patient volume:** a total of 229 patients were seen in the trauma resuscitation room over the study period of ten weeks, 162 patients in the first five weeks before total lockdown and 67 patients during the five weeks of total lockdown. This represents 41.35% reduction in the patient volume during the total lockdown. This reduction in the number of patients is expected in view of the restriction of movement imposed by the Nigerian government to curtail the spread of COVID-19. This is very similar to the findings in the United Kingdom where there was observed reduction of emergency department presentations by up to 30% [19] and acute orthopaedic trauma referrals by up to 34% [23]. This is also similar to other studies in Europe [20] and in Asia where a tremendous reduction of 75% in trauma emergency department footfall was reported [24].

**Socio-demographic characteristics:** males suffered more injuries than females during both periods of the study. This is a general pattern of trauma epidemiology globally and was not affected by the lockdown. The modal age group among the studied population during the two periods was 21 to 30 years followed by 31 to 40 years. These age groups represent the most active age groups who are more frequently involved in trauma. This is in line with the known fact that trauma most frequently affects the younger population and has remained the commonest cause of death in the same group [1].

**Duration and mechanism of injury:** more patients presented within two hours before lockdown while more patients presented after two hours during the lockdown period. This delayed presentation during the lockdown period may be due to multiple security check points in an attempt to enforce movement restriction. This observed difference is however, not statistically significant. The predominant mechanism of injury before and during total lockdown remained motor vehicular crash (MVC). This is followed by motorbike crash (MBC) before lockdown and penetrating assault during the total lockdown. MVC reduced from 37.65% before lockdown to 23.88% during the lockdown. The reason is obvious as fewer vehicles were allowed to move during the lockdown. This reduction in MVC was also reported in the Spanish association of surgeons´ document [25]. The proportion of penetrating assault increased from 6.2% before lockdown to 17.9% during lockdown. This higher prevalence of penetrating assault during the total lockdown may be partly due to the relative increase in crime rate occasioned by economic downturn during the lockdown.

**Referral status, mode of transportation and location of injury:** in the pre-lockdown period, only about 32.72% of patients were referred while in the lockdown period, more patients (53.72%) were referred. This may be related to increased presentation to nearby local health facilities due to restriction of movement. The predominant modes of transportation during both periods under study were private and security vehicles while commercial vehicles were least used. However, the proportion of patients who were transported in ambulance was significantly higher during the total lockdown period. This may not be unconnected to easy access at multiple security check points during the total lockdown. While majority of the injuries happened in the Abuja city before the lockdown, more occurred in the suburb during the total lockdown. This is understandable since majority of the population reside in the suburb where relative activities continued coupled with inadequate enforcement in those areas during the lockdown. The injuries that happened outside Abuja were minimal due to the interstate travel ban in place. When road related injuries are excluded, majority of the injuries (25%) happened at home during the lockdown due to sit at home orders, none happened in recreation centres which were closed during this period and only about 7% happened at workplace due to work from home order for non-essential workers.

**Diagnosis and injury severity:** in the pre-lockdown period, the most frequent diagnoses were soft tissue injuries, traumatic brain injuries and fractures/dislocations. During the lockdown, they were traumatic brain injuries, fractures/dislocations and polytrauma. This finding relatively reflects the usual injuries and was most likely not impacted by the lockdown. However, more severe injuries were seen before the lockdown than during the total lockdown. This observed difference in severity is statistically significant and may be attributed to more busy roads with no restrictions in the pre-lockdown era.

**Disposition from the resuscitation room:** the observed reduction of ward admissions, surgical interventions and patients leaving against medical advice (LAMA) from the resuscitation room during the lockdown probably mirrored the differences in the patient volume. While equal proportion of patients was admitted into the intensive care unit in both periods, more patients were admitted into the burns unit during the lockdown period. This later finding was due to burns from cooking gas and scald heightened by stay-at-home order. Finally, no patient died in the resuscitation room during the lockdown period. All the deaths recorded over the study period occurred before the lockdown period. This reflects the severity of injuries which were more in the pre lockdown period as discussed earlier.

**Limitations:** small sample size due to limited period of the lockdown is seen as one of the major limitations of this study. In addition, comparing the lockdown period with a similar calendar period in the previous year would have given a more objective analysis.

## Conclusion

The COVID-19 lockdown in Abuja Nigeria significantly reduced the total volume of acute trauma patients. There was a noticeable increase in referred cases and penetrating assault during the lockdown. Reduction in causes of severe injuries translated to reduced mortality in our centre. Efforts should be directed at sustaining access to acute trauma care in all circumstances to reduce preventable trauma deaths.

### What is known about this topic

- COVID-19 pandemic has affected the processes and the procedures in the management of acute trauma patients;
- COVID-19 lockdown was associated with reduction in trauma volume in different parts of the world.

### What this study adds

- Despite reduction in the overall acute trauma volume, COVID-19 lockdown was associated with a relative increase in subset of acute trauma patients referred to the trauma centre; in a setting with a hitherto poor referral system;
- COVID-19 lockdown in Abuja Nigeria was associated with less severe injuries and less mortality in the emergency room among acute trauma patients;
- The study will serve as a background knowledge in this subject matter, to stimulate further research in the future.
